# Priority Populations Toolkits: Enhancing researcher readiness to work with priority populations

**DOI:** 10.1017/cts.2019.436

**Published:** 2019-12-26

**Authors:** Kevin Rak, Alicia K. Matthews, Gabriela Peña, Wendy Choure, Raymond A. Ruiz, Sandra Morales, Amparo Castillo, Jackie Soo, Emily E. Anderson

**Affiliations:** 1 Center for Clinical and Translational Science, University of Illinois at Chicago, Chicago, IL, USA; 2 College of Nursing, University of Illinois at Chicago, Chicago, IL, USA; 3 Jane Adams College of Social Work, University of Illinois at Chicago, Chicago, IL, USA; 4 Loyola University Chicago Stritch School of Medicine, Maywood, IL, USA

**Keywords:** Toolkits, priority populations, health disparities, community engagement, CTSA

## Abstract

The National Center for Advancing Translational Sciences has called for more comprehensive research with priority populations to reduce disparities and for the development of additional resources to assist researchers in implementing these recommendations. Here we report the development and initial evaluation of five Priority Populations Toolkits, which are resources developed by the University of Illinois Center for Clinical and Translational Science to meet these goals. Three aims guide the content: increasing knowledge, facilitating communication, and improving research design. Materials were curated from scientific literature reviews and Internet searches and revised iteratively. Analytics and user surveys provide information about usage. In 22 months, 387 unique users accessed the toolkits. The top reason for usage was to improve research recruitment. Comprehensive toolkits for working with priority populations show promising potential for increasing knowledge and readiness to work with underrepresented populations. Further toolkit development and evaluation of effectiveness are warranted.

## Introduction and Background

Despite spending more than any other country on health care per person and as a percentage of GDP [1], the USA continues to have worse health outcomes compared to most industrialized nations [2]. These outcomes are even worse for certain segments of the population. Healthy People 2020 is a government initiative to improve population health and identifies several sociodemographic factors that are associated with poor health outcomes. These include race and ethnicity, gender, sexual identity and orientation, disability status or special health-care needs, and geographic location. For example, healthy life expectancy at age 25 is lower for Hispanics and non-Hispanic Black adults than it is for non-Hispanic Whites [3]. Additionally, lesbian, gay, and bisexual (LGB) populations are at increased risk for anxiety and depression, and LGB youth are 2–3 times more likely to attempt suicide than heterosexual youth [4]. Within the Department of Health and Human Services, two terms are used to describe these health disparity groups. The Agency for Healthcare Research and Quality uses “Priority Populations,”[5] while the Office of Special Populations in the National Institute on Aging uses “Special Populations.”[6] Feedback from people in the populations and subject matter experts suggests the term “priority populations” is the more acceptable term and will be used in this manuscript.

In addition to facing health disparities, priority populations are underrepresented in research. Clinical trial participation rates for Black and Hispanic cancer patients are substantially lower than for white patients;[7] this pattern is the same for other priority populations such as youth[8] and those without regular access to a health-care provider [9]. Researchers often fail to ensure that their studies are inclusive. A systematic review of lesbian, gay, bisexual, and transgender (LGBT) research suggests that researchers pay insufficient attention to recruiting LGBT participants or developing targeted interventions for this population [10]. Similarly, many studies’ eligibility criteria exclude the participation of people with disabilities, even when this exclusion is not scientifically or ethically justified [11]. From the perspective of priority populations, their members may be reluctant to participate in research because of mistrust, competing demands of time, unintended outcomes, lack of access to information, stigma, health insurance, and legal status concerns [12]. Unless data are gathered to ensure research findings are generalizable to these groups, it will be difficult to make progress on reducing disparities. Therefore, efforts are needed to improve recruitment and retention of research participants from priority populations.

Not all investigators have adequate training or background to effectively engage members of priority populations in research [13]. The Community Engagement Advisory Board (CEAB) at the University of Illinois at Chicago (UIC) has advised dozens of researchers on working with priority populations. The CEAB has identified several barriers to effective engagement with priority populations. One recurring issue is poor communication; investigators use jargon or withhold key information about their research plans from community partners. Additionally, some investigators have not developed the basic knowledge of a community or the cultural humility needed to work with priority populations. Power differentials represent another issue: often, researchers hold most of the decision-making power and get the benefits of the research, such as grant funding and the opportunity to publish. Community organizations and residents can therefore feel frustrated, leading to a reduced likelihood of participating in future research activities [14]. Highlighting these issues and providing guidance on how to resolve them is a crucial step in preparing investigators for research with priority populations. Resources for investigators to implement these changes are increasing but have not had sufficiently comprehensive content.

Two National Center for Advancing Translational Sciences goals point to the need for increased involvement of specific priority populations: Goal Two, “Engage patients and communities in every phase of the translational process,” and Goal Three, “Promote the integration of special and underserved populations in translational research across the human lifespan [15].” In response to these goals and challenges related to researcher readiness, the Community Engagement and Collaboration (CEC) core at the UIC’s Center for Clinical and Translational Science (CCTS) has developed a series of Priority Populations Toolkits. The toolkits’ objective is to provide researchers with the foundational knowledge and resources to increase their knowledge and readiness to effectively engage with specific priority populations. The purpose of this manuscript is to describe the development, content, dissemination, utilization, and initial evaluation of the Priority Populations Toolkits.

## Materials and Methods

### Guiding Principles

A range of community engagement tools and resources, often referred to as toolkits, have been developed to increase the involvement of community members in the design, implementation, and evaluation of a range of health-related initiatives. The Trial Innovation Network houses dozens of toolkits and other resources in its Recruitment & Retention Toolbox [16]. Informed by principles of community engagement, toolkits seek to provide guidelines to facilitating community engagement related to project-specific goals. A strength of existing toolkits is that they are broadly applicable to many different populations. However, a primary weakness is that they are often limited to information about specific endpoints such as recruitment, they are ahistorical, that is, they do not provide the historical background underlying many of the barriers to engaging specific communities in research, and they do not cover the range of information needed to increase the methodological skills and competencies of researchers.

The Priority Population Toolkits were developed to provide researchers with the knowledge, skills, and attributes (KSA) necessary for beginning community-engaged research with minority populations. The KSA Framework of Competency describes the prerequisite components to competent action. Knowledge refers to information that must be learned, skills are tasks that must be practiced in order to master, and attributes are personal qualities that must be developed if not already embodied. Each domain is necessary to achieve outstanding performance [17].

Following a review of the community engagement literature, members of the UIC CEAB were convened for a focus group to gather their insights on community engagement. Participants were selected who had significant experience conducting community-engaged research, living as members of priority populations, and/or advising researchers on conducting community-engaged research. Their advice to researchers that emerged from the focus group was categorized into knowledge, skills, and attitudes and published in a previous paper [14]. This framework was chosen because it operationalized existing principles of community engagement. Key elements of it are especially instructive. Under knowledge, Point 2 discusses the need to respect the issues facing the community of interest, including their sociopolitical history; this informed our first aim (below). A vital skill is Point 3, which states that researchers should demonstrate openness to community input via a collaborative approach; this guides our second aim. Finally, Point 7, under attitudes, discusses the need to value community input into the scientific process. This point informs our third aim. See Table 1 for the full framework.

**Table 1.** Knowledge, skills, and attitudes for community-engaged research

Knowledge
Articulate a community-centered focus and benefit of the research project and goals. Demonstrate respect and sensitivity for the issues facing the community one wants to engage with by effective communication and knowledge of the sociopolitical history of the group in the USA and locally.
Skills
Demonstrate openness to community input, perspectives, and priorities by establishing a collaborative team-based approach. Demonstrate cultural sensitivity, competency, and ability to engage across differences related to race/ethnicity, class, gender, sexual orientation, and immigration status, to name just a few.
Attitudes
Appreciate the importance of maintaining a presence and leadership in community engagement activities with community-based organizations and community members. Value equitable partnerships exemplified by equal power sharing, decision making, resource allocation, and costs associated with the research. Acknowledge and value community partner intellectual contributions to the scientific process. Value community capacity building as part of the key research outcomes and deliverables.

The toolkits include both frameworks for understanding issues relevant to each priority population as well as practical tools that can be used to prepare for and execute research projects. To accommodate investigators’ limited time, each toolkit provides a comprehensive introduction to working with each of the five specific priority populations. However, no toolkit can contain all the information an investigator needs to be successful in working with any specific population. As such, the toolkits and the website^1^ on which they are housed have connections to other resources, such as CCTS consultation services and other local and national Clinical and Translational Science Award (CTSA) resources, which can further increase knowledge and readiness to engage with priority populations.

### Developing Toolkits

To date, five toolkits have been developed and released: LGBT, Urban Youth, Hispanic and Latino/Latina, African American, and Disabled Populations. Toolkits specific to Asian Americans, Non-Native English Speakers, and Older Adults are planned for future development. These populations were selected because they are major populations in UIC’s surrounding catchment areas and resources are available at UIC to support research with these populations. Toolkits are hosted on the CCTS website.

Developing each toolkit was a multistage process. Each toolkit follows a similar outline, with 12 sections guided by three overarching aims, described below. Materials and resources are drawn from the scientific literature, UIC resources, materials developed by other CTSAs, and community resources. A draft of each toolkit was prepared by one staff member, then is sent to team members for review. This seven-person team includes diversity in academic achievement (three members hold PhDs, one holds a MD, several hold various master’s degrees), racial and ethnic background (three identify as Latina/Latinx, two identify as African American or Black, two identify as White), and sexual orientation and gender identity (one identifies their gender as nonbinary, another identifies sexuality as gay). Toolkits were reviewed by this team, along with relevant community stakeholders and content experts, including UIC’s CEAB. The feedback led to refinements of the language and content. Iterative changes were made until the toolkits were finalized.

## Toolkit Content

Each toolkit contains 12 sections, based on three aims.

### Aim 1. To Increase Knowledge of and Familiarity with Priority Populations

Knowledge and familiarity with the priority population forms an essential foundation for investigators to engage with a new population or community. Sections related to this aim include:

#### Historical and current issues related to the priority population

Learning essential aspects of a population’s history and current situation allows for a more holistic understanding of the community. For example, a researcher hoping to work with people with disabilities who does not know about how the researchers in the Willowbrook Study used coercion to get children with disabilities into their study may have a difficult time getting buy-in and support from that population [18]. This section provides context by discussing the history of the population in the USA and issues associated with participation in scientific research. Better understanding of the history and current issues related to research with the population helps researchers anticipate potential community concerns about research participation and proactively work to ameliorate those concerns.

#### National and local data

Data, particularly health outcomes data, play a significant role in forming research questions and developing hypotheses. For researchers unfamiliar with a population, however, they may not know where to find population-specific data. Information provided in this section includes links to various data sources on the population’s health and demographic characteristics, both nationally and specific to Chicago and the greater metropolitan area. Accessing population-specific local and national data has multiple benefits. Researchers can review existing data to identify issues they would like to investigate further. Alternatively, those with existing research questions can quickly find data to inform their hypotheses. Further, secondary analysis of these data can be useful as pilot data for grant applications or help illustrate the scope of a local health issue to community-based organizations when developing partnerships.

#### Community engagement resources

Many organizations are already working with the population of interest. These organizations have deep knowledge of the population and can be key resources for investigators. In this section, investigators can find lists of community-based organizations who focus on the population, on a national level and in the Chicago area. While this list is not exhaustive, it provides investigators a sense of the organizations working with the relevant population and helps them identify potential community partners.

#### Local researchers and centers working on the issue

Learning about investigators and research centers working with a local priority population is necessary so that researchers can avoid duplicating what has already been done and instead build upon existing research. This section lists faculty and centers at UIC, Northwestern University, and the University of Chicago who study the population. Faculty and centers were identified by searching several outlets: searching the CTSA and individual college websites, reaching out to campus administrators, and searching sites like clinicaltrials.gov. This procedure can be followed to identify local resources in other areas. While the list is not exhaustive, it gives investigators who are new to working with a specific priority population a sense of who is active in the field, helping them to identify potential collaborators or mentors.

### Aim 2. Facilitate Effective Communication and Positive Interactions with Priority Populations

Communicating effectively with the priority population is a vital skill that investigators and their teams must use to build partnerships with community stakeholders. The following sections provide information and resources needed to appropriately engage with individuals and organizations from the priority population.

#### Recruitment and retention best practices

This section describes population-specific suggestions for improving recruitment, retention, and community engagement. A range of approaches to facilitating connection with communities (including community-based participatory research and community engaged research) are described and encouraged because of the potential benefits they offer [19] and the emphasis organizations like the Clinical and Translational Science Awards Program [15]. Specific examples of best practices in community engagement include a discussion of the role gatekeepers play in recruiting urban youth and the need to be careful about asking about documentation status while working with Hispanic and Latino/Latina populations. Relevant literature discussing community involvement in research is presented, as are various options available to investigators, including community-based participatory research and community-engaged research.

#### Recruitment templates

Often, the first point of contact with potential participants is recruitment material, such as flyers or listserv emails. This section gives examples of flyers, websites, and terminology that can be used with a priority population. The flyers typically come from studies that have received IRB approval, showing investigators exactly what they need to include to obtain approval for their own outreach materials.

#### Community stakeholder involvement

Community partnerships are essential to appropriate engagement. Community organizations staff by and/or working with the priority population are the audience for this section, which presents information for individuals and organizations to understand research and the research process, along with resources showing how to get involved in clinical trials and other research, including resources at our institution. This information can be tailored to include local resources of other CTSAs as well.

#### Team readiness to work with priority populations

Investigators usually rely on a research team to handle most interactions with participants. All research team members should have the knowledge, skills, and attitude necessary to work with the population. This section provides resources to help research teams conduct an initial assessment of their readiness to engage with the population. These include general assessments measuring cultural humility and implicit bias, plus specific assessments about perceptions of the population being discussed. Investigators can use these tools to ensure their staff have the necessary knowledge, skills, and resources to work with the population.

### Aim 3. To Promote Sensitive, Responsive, and Effective Research Methodologies and Processes

Sound knowledge and effective communication are insufficient without good research design. Research with underrepresented groups should be sensitive and responsive to their unique needs, as well as effective. The following sections provide guidance on establishing collaborative research methodologies and processes.

#### Health and research practice

Evidence-based practices for health and research that have been adapted to the unique needs of priority populations should be a guide for practice and a starting point for research. An example is the management of high blood pressure in adults, which includes recommendations specific to African Americans [20]. This section presents some of these recommendations along with portals for finding other evidence-based practices.

#### Ethical and regulatory issues

Working with priority populations often entails special ethical and regulatory considerations. This section describes those ethical challenges investigators face when researching the population. For example, research with minors normally necessitates obtaining parental consent. Doing so, however, is potentially harmful when the youth are LGBT, particularly if they are not out to their parents/guardians. Guidance is presented, including information that can be shared with one’s IRB. It also introduces regulatory requirements that must be followed in order to get approval from the IRB. These discussions prime researchers to refine their methods earlier, reducing the likelihood of problems and delays later in the process.

#### Measurement instruments

Validated tools may have only been tested with racial and ethnic majorities, people without disabilities, etc. The tools listed in this section, however, have been tested with the population being discussed and have demonstrated good psychometric properties.

#### Program announcements for grants

Funding is essential to turn an idea for a project into reality. This section lists grant opportunities to fund research with the population. Grants listed are selected because they specifically provide funding for research. They are updated periodically to ensure that they are still active.

## Dissemination and Evaluation Methods

### Disseminating Toolkits

Systematic efforts were made to disseminate the toolkits. Each of the Priority Population Toolkits has been shared via local and national listservs. They have been posted to the Recruitment & Retention Toolbox section of the Trial Innovation Network website^2^ in addition to the UIC website. Finally, they have been featured at local workshops, and a poster describing early development was shared at the Association for Clinical and Translational Science 2018 conference.

### Evaluating Toolkits

The CCTS website uses Google Analytics to collect general usage information. In addition, the Community Engagement & Collaboration Core has worked with the Evaluation and Tracking core to develop a two-phase strategy for eliciting feedback. In the first phase, users provide information about their organization, role, and purpose for downloading the toolkit and are asked to indicate if they agree to be contacted at a later time to provide information regarding toolkit use. In phase two, the CCTS Evaluation and Tracking core followed up with users who agreed to be contacted and asked about their toolkit usage, satisfaction with the toolkit, and how it has informed their research methods or approaches (Table 2). After feedback from peers at other institutions, a question will be added soliciting suggestions on how to improve the toolkits. The existing questionnaire was sent to these users via Qualtrics, along with multiple reminder messages.

**Table 2.** User questionnaire

Phase 1
Are you part of a Clinical and Translational Science Award (CTSA) Institution (Clinical and Translational Science Award) Institution? Yes/No If yes, what is your institution? University of Illinois at Chicago Northwestern University University of Chicago Other If no, are you based in the Chicago metropolitan area? Yes/No
With which role do you most closely identify? (check all that apply) Community member/partner Researcher/academic Practitioner (doctor, social worker) Student Administrator Journalist, blogger Other (if other, please define)
Which toolkit(s) did you download? African American Hispanic Latino/Latina LGBT Urban Youth People with Disabilities
How will you use the information? (check all that apply) Grant application for research Research recruitment Clinical interactions with patients General outreach Training/teaching (for example, curriculum development) Other (if other, please define)
May we contact you with future toolkit updates? Yes/No
May we contact you regarding your use of the toolkit? Yes/No Name (last, first) Email
Phase 2
How did you hear about the Target Populations Toolkits? Colleague, peer, or mentor. Name of person who referred you: ————————— Faculty or student orientation CCTS eBlast or event email CCTS website Department provided information Other: ——————————
Have you been able to use the Target Populations Toolkit yet? Yes/No: If No → Go to Question 3, Else Skip to 4
Why haven’t been able to use the Target Populations Toolkit as of now? Study under development/Idea generation phase Will be used at a later stage in our study The toolkit is not what we expected The toolkit will not meet our needs Other:——————————— → end of survey
Overall, how satisfied were you with the toolkit? Very satisfied/Somewhat satisfied/Neither satisfied nor dissatisfied/Somewhat dissatisfied/Very dissatisfied
What were the components of the toolkit that you found most helpful? [Open-ended]
What were the components of the toolkit that you found least helpful? [Open-ended]
Can you give any specific outcomes that resulted from your use of the toolkit (e.g.: changed participant outreach or recruitment, developed new training, or teaching programs, etc.)? [Open-ended]
If you are using the toolkit for research purposes, what is the current status of your research? Under development Awaiting IRB approval In data collection Completed Not yet implemented Not being used for research purposes Other:—————
Do you anticipate any of the following benefits to result from the research or other work for which you used the toolkit? If yes, please describe the specific anticipated benefits. [Open-ended sections for all] Clinical and medical benefits, such as new diagnostic, investigative, or therapeutic procedures, biomedical technologies, or drugs. Community and public health benefits, such as increased community health services, improved health-care delivery, or new public health practices. Economic benefits, such as patents or research on cost effectiveness or cost savings. Policy and legislative benefits, such as new policies, legislation, or standards, or contributing to scientific research reports.
Do you anticipate using any of the other Target Populations Toolkits? Yes/No If Yes, Which ones: ————————— If No, Why not?——————————

## Results

### Utilization and Initial Evaluation of the Toolkits

We gathered several types of data to gauge the reach of the toolkits, in terms of number of users, location of users, and users’ demographic information. Toolkit usage data come from the CCTS and TIN websites. Between October 2017, when the first toolkit section was posted, and July 2019, when 387 unique users accessed the toolkits. The largest number of users (57.0%, *n* = 221) has come from the Chicago area. Substantial portions of other users have come from the areas of New York (4.64%, *n* = 18), Washington, DC (4.38%, *n* = 17), and Raleigh-Durham (3.35%, *n* = 13); remaining users (30.5%, *n* = 118) have come from other areas in the USA and worldwide.

We analyzed download data for the 87 users who provided their contact and demographic information shown in Table 2. Most individuals who downloaded a toolkit (66.7%, *n* = 58) came from a CTSA program. As shown in Table 3, the most common role was researcher or academic (67.8%, *n* = 59). The most common stated reason for download was for research recruitment (48.3%, *n* = 42), followed by training and teaching (29.9%, *n* = 26); further purposes are shown in Table 4.

**Table 3.** User roles

Role	*n*	%
Researcher/academic	59	67.8
Student	15	17.2
Practitioner (doctor, social worker)	8	9.2
Administrator	8	9.2
Other	8	9.2
Community member/partner	7	8.0
Journalist/blogger	0	0.0

**Table 4.** Purpose for download

Purpose	*n*	%
Research recruitment	42	48.3
Training/teaching	26	29.9
Other	23	26.4
General outreach	20	23.0
Grant application for research	13	14.9
Clinical interactions with patients	7	8.0

### User Follow-Up Survey Responses

The survey shown in the second part of Table 2 was sent to the 66 users who consented to be contacted about their use of the toolkits. Of these, 19 (28.8%) responded to the survey request. Most respondents (*n* = 15, 78.9%) stated they had not had an opportunity to use the toolkits in their research yet. About half (*n* = 8, 53.3%) of these indicated they will use the toolkits in a later stage of the study or that their study was in development. The remainder offered other responses: planning to offer it as a resource to other researchers, not currently conducting research themselves, or not having had the opportunity to discuss the toolkit with their principal investigator. Four respondents indicated they had used the toolkits. Three of them said they were very satisfied, and one said somewhat satisfied. They indicated the most helpful areas were examples of language that could be used in recruitment, marketing tools, and information about incentives for participation. Future surveying of users should be able to give a larger sample of respondents who have used the toolkits.

## Discussion

### The Toolkits’ Place in Enhancing Researcher Readiness, Locally and Nationwide

Priority Population Toolkits provide information that aids researchers in becoming acquainted with relevant issues and data associated with each of the five priority populations, helping them to begin their research projects. However, no online resource can make investigators experts at working with the population, nor will it answer all the questions they will have. CTSAs must provide other resources and supports to investigators intending to work with priority populations. At UIC, the CEC core provides a variety of resources to meet investigators’ needs. These include a symposium series on working with priority populations, individual consultations with expert faculty advisors, and consultations with the CEAB. Together, these resources can propel novice investigators into becoming adept at working with priority populations. CEC tracks use of these resources and will be assessing long-term impact of its engagement in future years.

In addition to providing investigators with a comprehensive array of information, the Priority Populations Toolkits serve as a model to CTSAs nationwide. The toolkits we have produced were selected because they are among the major underserved populations in the Chicago metropolitan area, and there are resources at UIC and other local institutions to which investigators can be connected. In other areas, different populations would be appropriate to feature in toolkits. The University of North Carolina – Chapel Hill had a consultation with CCTS staff on the creation of the toolkits so they could create one on rural populations, a priority population for that university.

### Challenges, Limitations, and Future Plans

Some challenges have emerged in creating the toolkits. The most significant is ensuring the information is accurate and updated. The toolkits are therefore reviewed every 6–12 months and edited as needed. An additional challenge is the several months needed to create each toolkit. However, initial results demonstrate the utility of the toolkits, suggesting creating them may be worthwhile.

Several limitations exist. The primary limitation is the low response rate of people who had downloaded and used the toolkits. The feedback they provided was helpful for our thinking but not generalizable to all users. In the future, the toolkits will have been available for longer, which both gives people who have already downloaded them more time to use the toolkits, and creates the opportunity for additional users. In the future, we also may offer a modest gift card or other incentives to encourage participation. This should allow us to have a sufficient sample size to draw meaningful conclusions. However, this paper was shared to provide a timely update to CTSAs nationwide.

Other limitations include that the toolkits were created at one institution. Another institution may have approached the topics differently. Also, the website analytics and user response surveys may not fully represent all users. Some users abandoned using the toolkits when presented with the initial request for their demographic information.

Future plans for the toolkits include finishing the series by adding toolkits for Older Adult, Asian American, Native American, and non-native English-speaking populations. Follow-up data will continue to be collected periodically to evaluate the toolkits’ effectiveness more robustly. The Research Toolkit for Community Organizations will be described elsewhere. Longer-term, UIC will share the toolkits through different channels, including webinars and creating an online, interactive version.

## Conclusion

Priority populations face dual disparities. Lack of investigator preparedness to work with these populations accounts for some of the gaps in data. To enhance investigator readiness to work with priority populations, the Priority Populations Toolkits provide an extensive introduction into the information and resources investigators need. The initial response has been positive and widespread, suggesting an appetite for this type of resource. Along with complementary resources, the toolkit can help investigators increase the diversity of their study populations, leading to better data and interventions to reduce health disparities.

This approach can serve as a model for other CTSAs to improve engagement, recruitment, and retention with priority populations. One strength is that the sections can be adapted to local conditions, both in terms of different priority populations and the research landscape in the spaces where CTSAs operate. This can foster a more equitable relationship between investigators and communities, creating a virtuous cycle of community involvement and participation in research.

## Footnotes

1 http://www.ccts.uic.edu/content/target-populations-toolkit.

2
https://trialinnovationnetwork.org/recruitment-retention-toolkit/?key-element=1681.
